# Lung cancer associated with cystic airspaces: current progress and future perspectives

**DOI:** 10.3389/or.2025.1615365

**Published:** 2025-09-24

**Authors:** Jiarui Wang, Jie Dai

**Affiliations:** Department of Thoracic Surgery, Shanghai Pulmonary Hospital, School of Medicine, Tongji University, Shanghai, China

**Keywords:** lung cancer, lung cancer associated with cystic airspaces, clinicopathological features, image characteristics, pathogenesis, prognosis

## Abstract

Lung cancer associated with cystic airspaces (LCCAs) is a distinct subtype of lung cancer defined by its unique radiological characteristics. It is increasing in prevalence but is often misdiagnosed. The constantly emerging radiological classification systems help characterize LCCAs and guide certain treatment methods. Compared to non-LCCAs, LCCAs are more likely to be associated with an invasive nature. The natural progression of LCCAs involves the thickening of cyst walls and the emergence of solid nodules, which are indicative of tumor progression. Despite their aggressive features, the overall prognosis of LCCAs was similar to non-LCCAs this review, we aim to systematically address the current understanding of LCCAs, including the epidemiology, radiologic classification, pathology, molecular characteristics, disease progression, and survival prognosis, highlighting the need for further research to standardize the diagnosis and treatment of LCCAs and to better understand their mechanisms of development.

## Introduction

Lung cancer is one of the most common and deadliest malignant tumors (1). Recently, lung cancer associated with cystic airspaces (LCCAs) has gradually emerged as a significant but potentially underappreciated subtype (2). LCCAs was first defined by Farooqi et al. as “lung cancers that about or are in the wall of cystic airspaces identified at CT, regardless of the pathology findings and irrespective of the presence of emphysema elsewhere in the lung” (3). “Cystic airspaces” were historically considered to be associated with benign diseases (4, 5). However, it was recently observed that certain lung cancers were associated with it (6). Womack first reported LCCAs in 1941 (7). Later, Anderson and Pierce introduced a series of radiological manifestations as thin-walled cysts in cases of bronchogenic carcinoma (8). Subsequently, multilocular cysts and emphysematous bullae have also been described as being associated with lung cancer afterward (9–11).

LCCAs was also denoted as “cystic lung cancer,” “lung cancer adjoining bullae,” and “pulmonary cavity nodules” in the past (12–14). This diversity of terminology reflected the lack of a unified understanding, leading to inconsistencies in research and confusion in clinical recognition. In the Nederlands-Leuvens Longkanker Screenings Onderzoek (NELSON) lung cancer screening trial, 22.7% of lesions presented as LCCAs that were missed or delayed (15, 16). The NELSON trial demonstrated that structured LDCT screening improved outcomes by 24%; however, patients with missed or delayed diagnoses could not benefit from these advantages, as disease progression during follow-up may compromise surgical eligibility and survival (17–19). Cystic airspaces lead to diagnostic challenges, such as difficulty in distinguishing them from benign lesions such as tuberculosis or fungal infections on imaging, and obtaining sufficient tissue samples from thin or irregular cyst walls is difficult (13, 20). In terms of treatment, the presence of cystic airspaces may complicate margin assessment and treatment response because of the lack of relative standards (21). Therefore, the diagnosis and management of LCCAs cannot simply rely on the standards for conventional lung cancer. It is essential to elucidate the epidemiology, clinical features, and progression patterns of LCCAs.

In this review, we aim to address these issues to advance the current understanding of LCCAs, facilitating early identification, providing opportunities for early treatment, and accurately assessing treatment outcomes.

## Epidemiology

The prevalence rates of LCCAs vary widely from 0.46% to 12.6%, largely reflecting differences in study design and populations (22–26). In the pre-existing cysts, the proportion of LCCAs reached 4.6% (22). The incidence of LCCAs is influenced by the frequency of CT scans and the number of patients screened, as well as the differences in the definition of LCCAs. Farooqi et al. reported that the detection rate of LCCAs in the baseline round of screening was 2%, whereas it increased to 12% in the annual rounds (3).

## Demographic characteristics

Similar to the demographic characteristics of lung cancer, LCCAs was frequently observed in middle-aged and elderly individuals. More than half of patients were male (27, 28). In most Asian studies, LCCAs predominantly affected male patients, whereas female patients represented the majority in Western cohorts, which contrasts with findings from subsolid nodules (SSNs), highlighting that LCCAs may not fully share the same demographic distribution as SSNs (29). The prevalence of smoking among these patients varied widely in different studies, ranging from 12.5% to 100% (30, 31) (Table 1).

**TABLE 1:** Demographic characteristics of LCCAs.

Demographic characteristics		Zhu, 2023 (26)	Shen, 2021 (32)	Jung, 2020 (33)	Toyokawa, 2018 (34)	Fintelmann, 2017 (28)	Iwama, 2016 (35)	Guo, 2016 (22)	Watanabe, 2016 (36)	Farooqi, 2012 (3)	Byrne, 2021 (37)	Ma, 2022 (31)
Cases, n		252	123	60	31	30	45	15	143	26	47	384
Proportion		265/2,093 (12.6%)	123/10,835 (1.13%)	60/1,971 (3%)	31/283 (11%)	30/2,954 (1.0%)	45/488 (9.2%)	15/3268 (0.46%)	143/3192 (4.48%)	26/706 (3.7%)	47/431 (10.9%)	
Sex, n(%)												
	Male	128 (50.8)	82 (66.7)	44 (73.3)	27 (87.1)	12 (40.0)	43 (95.6)	12 (80)	97 (67.8)	13 (50)	20 (42.6)	236 (61.5)
	Female	124 (49.2)	41 (33.3)	16 (26.7)	4 (12.6)	18 (60.0)	2 (4.4)	3 (20)	46 (32.2)	13 (50)	27 (57.4)	148 (38.5)
Age (mean or median)^a^		58.0 ± 11.1	61 (54–67)		69 (29–85)	66.2 (44–84)	63.6 (60.5–66.8)	58 (39–77)	63 (26–82)	65.15 ± 13.25	69 (65–74)	58.2 ± 10.8
Smoking history, n (%)												
	Ever			37 (61.7)	30 (96.8)	29 (96.7)			95 (66.4)	26 (100.0)	39 (83)	48 (12.5)
	Never			23 (38.3)	1 (3.2)	1 (3.3)			48 (33.6)		8 (17)	336 (87.5)
Number of packs per year (mean or median)^a^						46 (15–110)	53.2 ± 9.2				45 (36–59)	

^a^ Data are presented as the mean ± standard deviation (SD) or the median (range).

## Radiologic classification

LCCAs can be observed on CT scans as either solitary or multiple cystic components, accompanied by air density shadows within.

### Maki–Mascalchi classification

**FIGURE 1:**
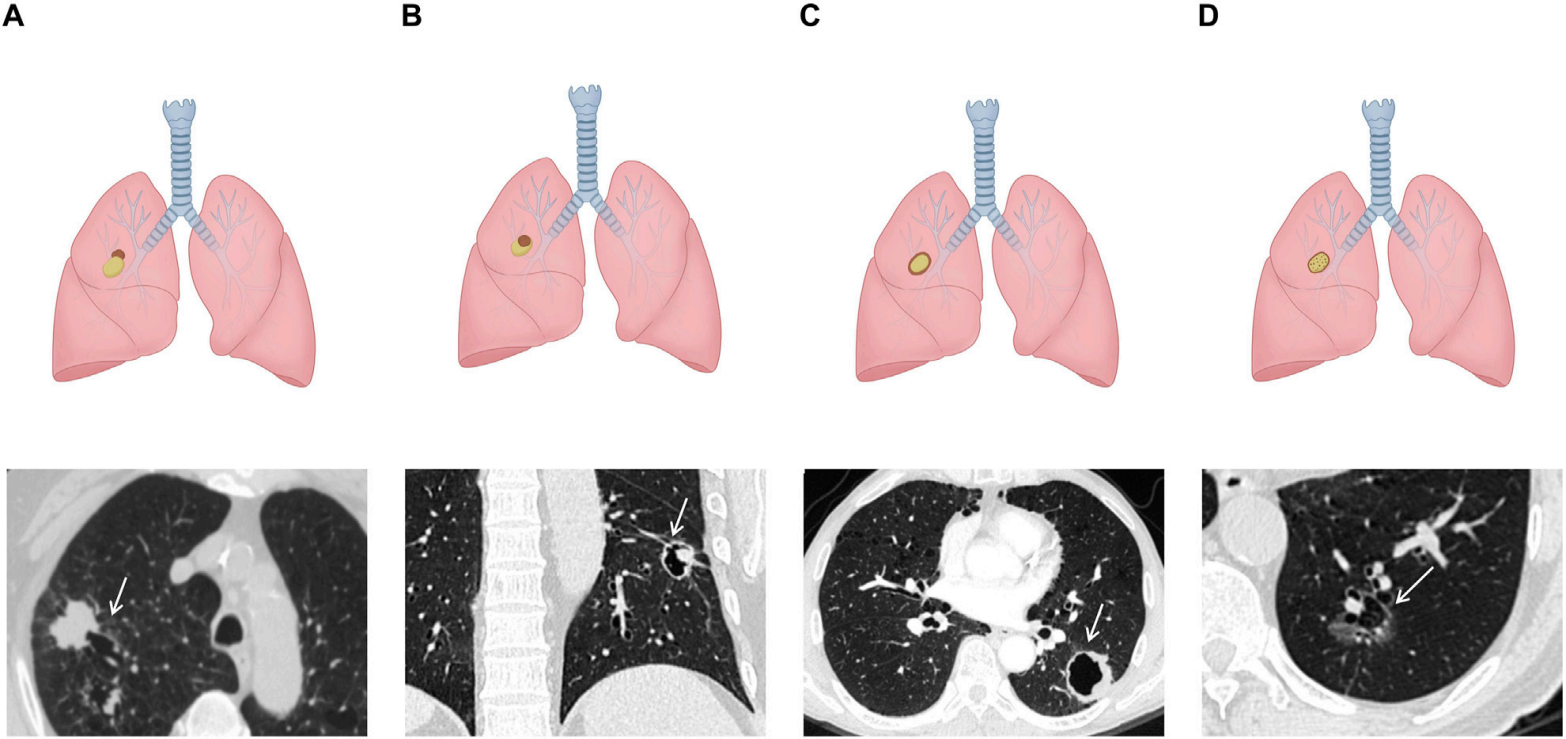
Schematic illustrations and CT images of Maki–Mascalchi classification: **(A)** type I: solid nodule protruding externally from the cystic cavity. **(B)** Type II: solid nodule protruding internally from the cystic cavity. **(C)** Type III: circumferential thickening of the cyst wall. **(D)** Type IV: soft tissue density intermixed within clusters of the cyst.

Initially, in 2006, Maki et al. proposed a classification system for LCCAs (38), which was subsequently refined by Mascalchi (39) (Figure 1). The classification categorized LCCAs into four subtypes based on their morphological characteristics: type I: nodule or mass extruding from the wall of the cystic airspace (Figure 1A); type II: nodule or mass confined within the cystic airspace (Figure 1B); type III: soft tissue density extending along the wall of the cystic airspace (Figure 1C); and type IV: soft tissue density intermixed within clusters of cystic airspaces (Figure 1D). Among them, type III was the most common type (39, 40).

### Fintelmann classification

**FIGURE 2:**
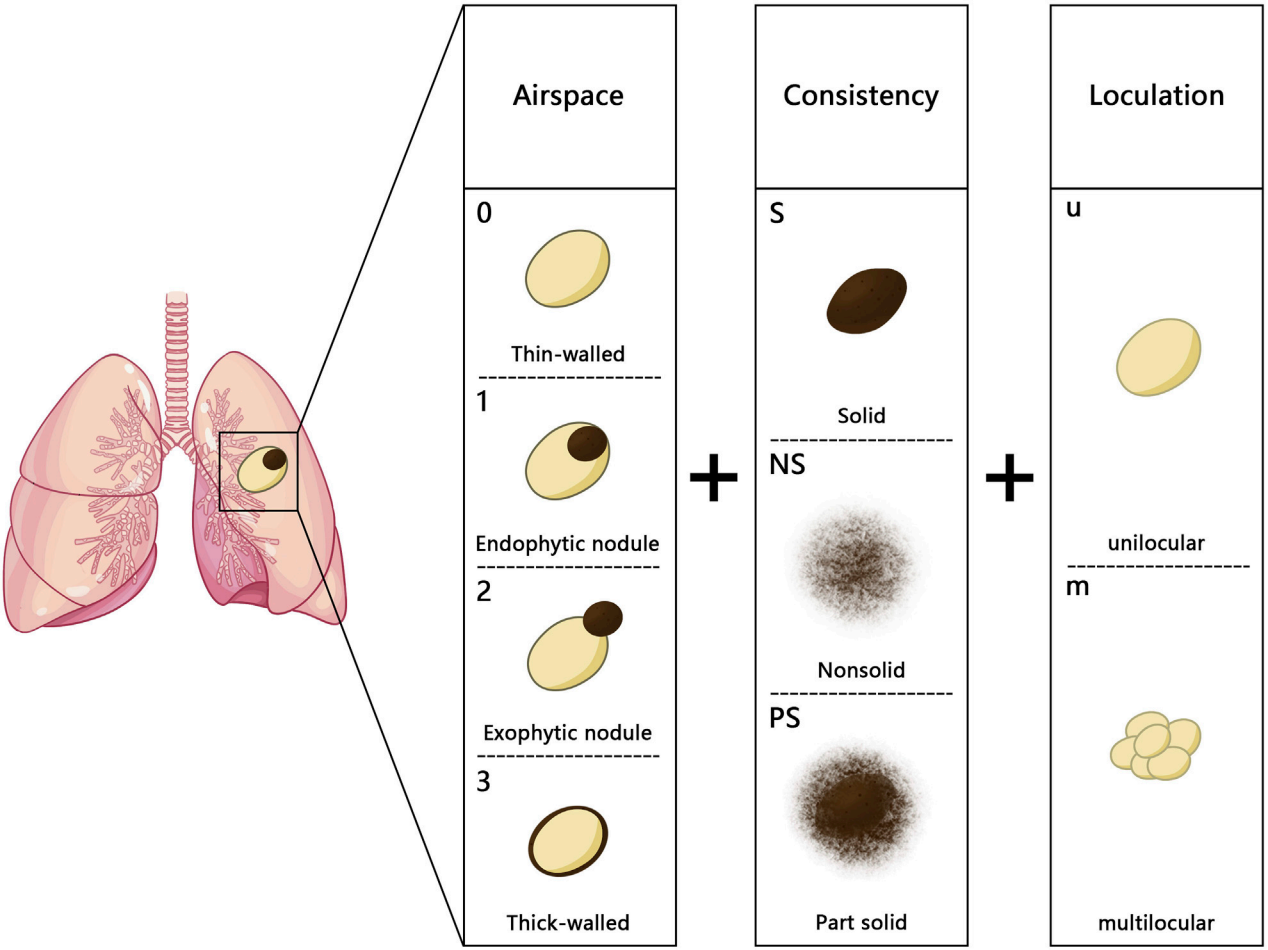
Fintelmann classification: the numbers (0–3) represent different types of the wall: 0. thin-walled cystic airspace with a maximum wall thickness of 1 mm, 1: endophytic nodule, 2: exophytic nodule, and 3: thick-walled with a wall thickness greater than 1 mm; the uppercase letters (S, NS, and PS) indicate the nodule density (S, solid; NS, nonsolid; PS, part solid); the lowercase letters (u and m) represent the loculation of the cystic airspaces (u, unilocular; m, multilocular).

In 2017, Fintelmann et al. introduced a new classification system based on the Maki–Mascalchi classification (28). The modified classification system included a numerical component, optional uppercase letters, and a lowercase letter to represent the wall, nodule, and cystic components. For the first time, this system also considered thin-walled cysts (with a maximum thickness of 1 mm) and ground glass nodules (Figure 2). This classification system was more accurate as it took the diverse manifestations of components of LCCAs into account. This classification method was primarily suitable for investigating the natural progression of LCCAs, but it was difficult to conduct clinical research due to the limited number of patients across different groups.

### SPH classification

**FIGURE 3:**
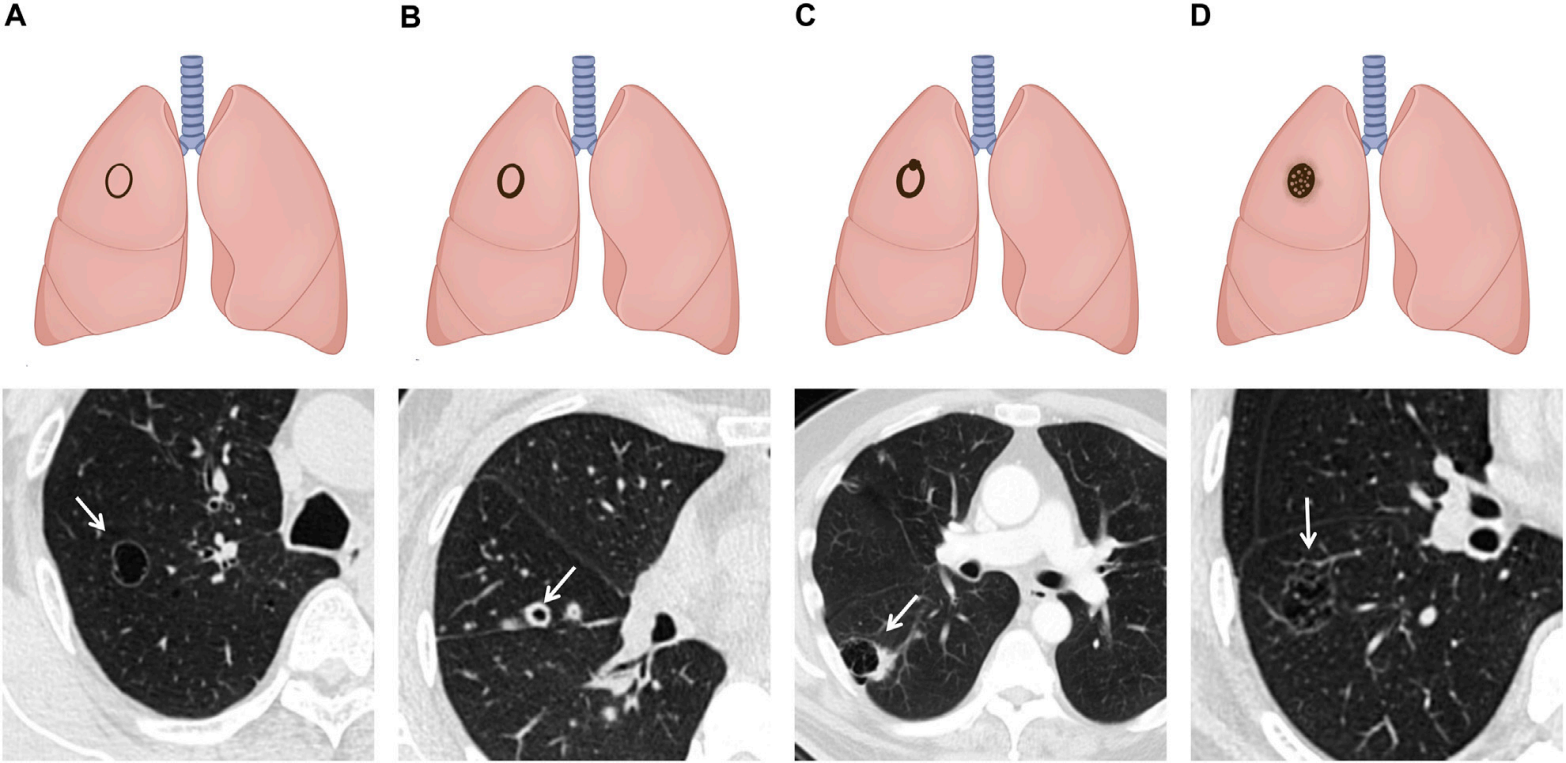
Schematic illustrations and CT images of SPH classification: **(A)** type I: thin-walled type: cyst wall thickness of less than 2mm. **(B)** Type II: thick-walled type: cyst wall thickness of more than 2 mm. **(C)** Type III: CWN type: a cystic airspace with a mural nodule that is either endophytic or exophytic. **(D)** Type IV: mixed type: tissue intermixed within clusters of multiple cysts.

Shen et al. introduced the SPH classification in 2019, distinguishing four types: type I (thin-walled type): cyst wall <2 mm; type II (thick-walled type): cyst wall >2 mm; type III (cystic airspace with mural nodule, CWN): a cystic airspace with a mural nodule that is either endophytic or exophytic; and type IV (mixed type): tissue intermixed within clusters of multiple cysts. It incorporated the thin-wall type and consolidated the Maki–Mascalchi types I and II into the cystic airspace with a mural nodule type (SPH type III) (41) (Figure 3). According to this classification, SPH type III accounted for a larger proportion (43/123, 35.0%), which was further confirmed by the research by Ma et al. (31). Because some previous studies excluded cases lacking sufficient evidence to demonstrate that the tumor originated from a pre-existing thin-walled cystic airspace, the above results may lead to an underestimation of the incidence of thin-wall-type LCCAs (39, 41).

### Jung classification

These classifications did not take into account the development of the ground glass opacities (GGOs) component of LCCAs. Recently, Jung et al. proposed a new classification system based on the evolution and development of LCCAs (33). The four subtypes distinguished GGOs, part-solid, and pure-solid LCCAs, linking CT findings with pathological progression (26). It was the first classification system that considered the proportion of solid components, which was in concordance to the differentiation of pulmonary nodules.

## Pathology

### Location and histology

The majority of LCCAs was in the peripheral site, accounting for approximately 70% of the cases, without the tendency of pulmonary lobar inclination (30). Adenocarcinoma accounted for more than 70% of LCCAs (31, 41), whereas fewer than 30% of patients had squamous cell carcinoma (32, 42, 43). The proportion of patients with LCCAs coexisting with emphysema was relatively high, with a reported rate of 73% (3) (Table 2).

**TABLE 2:** Clinical characteristics of LCCAs.

Clinical characteristics		Zheng, 2024 (44), n (%)	Ma, 2022 (31), n (%)	Shen, 2019 (41), n (%)	Tan, 2019 (45), n (%)	Haider, 2019 (30), n (%)	Shinohara, 2018 (14), n (%)	Fintelmann, 2017 (28), n (%)	Iwama, 2016 (35), n (%)	Guo, 2016 (22), n (%)	Mascalchi, 2015 (39), n (%)	Farooqi, 2012 (3), n (%)	Byrne, 2021 (37), n (%)
Stage	I	154 (92)			67 (63.2)		34 (65.4)	18 (60.0)	16 (35.6)	9 (60)	12 (50.0)	21 (80.8)	
	II	7 (4)			12 (11.3)		14 (26.9)	5 (16.7)	2 (4.4)	2 (13.3)	3 (12.5)	2 (7.7)	
	III	7 (4)			11 (10.4)		4 (7.7)	2 (6.7)	10 (22.2)	3 (20)	4 (16.7)	3 (11.5)	
	IV				16 (15.1)		0 (0)	5 (16.7)	17 (37.8)	1 (6.7)	5 (20.8)		
Emphysema				38 (30.9)	5 (4.7)	5 (45.5)		29 (96.7)				19 (73.1)	
Lobe location	RUL				25 (23.6)	1 (9.1)	36 (69.2)^a^	6 (20.0)	32 (71.1)	4 (26.7)	10 (41.7)	7 (26.9)	13 (26.5)
	RLL				27 (25.5)	4 (36.4)	15 (28.9)^b^	8 (26.7)	12 (26.7)	3 (20)	7 (29.2)	5 (19.2)	16 (32.7)
	RML				10 (9.4)	2 (18.2)	1 (1.9)	3 (10.0)	1 (2.2)	1 (6.7)	1 (4.2)	2 (7.7)	2 (4.1)
	LUL				23 (21.7)	3 (27.3)		7 (23.3)		3 (20)	3 (12.5)	9 (34.6)	10 (20.4)
	LLL				21 (19.8)	1 (9.1)		6 (20.0)		4 (26.7)	3 (12.5)	3 (11.5)	8 (16.3)
Location	Peripheral		289 (75.3)			7 (63.6)		24 (80.0)		15 (100)	4 (16.7)		
	Central		95 (24.7)			4 (36.4)		6 (20.0)			20 (83.7)		
Histology	SCC	8 (5)	115 (29.9)	6 (4.9)	7 (7.5)	2 (18.2)	18 (34.6)	4 (13.3)	13 (28.9)	2 (13.3)	7 (29.2)	1 (3.8)	9 (18.4)
	Adenocarcinoma	155 (94)	237 (61.7)	117 (95.1)	81 (87.1)	9 (81.8)	26 (50.0)	24 (80.0)	23 (51.1)	11 (73.3)	17 (70.8)	23 (88.5)	39 (79.6)
	Others	2 (1)	5 (1.3)		5 (4.7)		8 (15.4)	2 (6.7)	4 (8.9)	2 (13.3)		2 (7.7)	1 (2.0)

RUL, right upper lobe; RLL, right lower lobe; RML, right middle lobe; LUL, left upper lobe; LLL, left lower lobe; SCC, squamous cell carcinoma.

^a^ Upper lobe from both the right and left sides.

^b^ Lower lobe from both the right and left sides.

### Pathological features

Compared to non-LCCAs, LCCAs was more likely to be associated with pleural and vascular invasion, as well as histological invasive subtypes such as papillary or solid component (34). SPH type III, solid wall, and large cystic airspaces were independently predictive of a poor differentiation of LCCAs, which may guide the extent of surgical resection (31). Multiple cystic airspaces, irregular shape of cystic airspaces, whole tumor size, and CT attenuation were identified as independent risk factors for the pathologic invasiveness of LCCAs, and a thicker wall was indicative of vascular and/or lymphatic invasion, suggesting a need for careful intraoperative evaluation (26, 36, 41). Regarding the LCCA subtypes, SPH type I represented an earlier stage of disease because it manifested as adenocarcinoma *in situ*, whereas patients with SPH type III or IV had more invasive characteristics. In addition, type III was identified as an independent risk factor for pathological invasion in LCCAs (41). The majority of type IV lesions exhibited lepidic, acinar, or papillary characteristics (32). Therefore, the thickness of the cyst wall, in conjunction with the presence of solid components, collectively determined the pathological invasiveness of tumors and provided actionable information for surgical planning and follow-up strategies.

Tan et al. found that an uneven wall on CT reflected the infiltration of tumor cells into normal tissues, whereas a wall nodule was indicative of tumor cell proliferation within the alveolar cavity. GGOs signified the expansion of tumor cells along the alveolar walls, irregular margins resulted from inward folding of tumor cells into fibrous tissue within cyst walls, and septations within the cyst might consist of fibrous tissue generated by tumor cells, bronchi, or blood vessels (45). Recognizing these imaging characteristics could help radiologists and surgeons predict pathological invasiveness preoperatively and optimize the extent of resection, thus directly linking CT findings with clinical decision-making.

### Tissue sampling

Notably, in terms of pathological specimens, conventional percutaneous and bronchoscopic biopsy had historically faced challenges in acquiring sufficient diagnostic samples of LCCAs due to the thinness of the cyst wall and the small number of obtainable cells (22). Moreover, some operators may be concerned that biopsies of lesions containing airspaces may result in tumor rupture and pneumothorax (46, 47). However, several studies showed that CT-guided core needle biopsy had a comparable safety in evaluating lesions associated with LCCAs as non-LCCAs (48, 49). In addition, CT-guided fine-needle aspiration biopsy and CT-guided percutaneous needle biopsy have also been shown to be effective and safe for cavitary or thin-walled lesions, providing additional options in challenging cases (50, 51). For patients who are surgical candidates, intraoperative tissue sampling or resection remains the most definitive and effective diagnostic approach.

## PET-CT

Consistent with the more aggressive pathological classification, LCCAs demonstrated higher FDG uptake. In the study regarding “emphysematous bullae-associated lung adenocarcinomas,” the SUVmax of cancer adjoining emphysematous bullae was significantly higher than that of cancer without emphysematous bullae (34). During the follow-up process, FDG uptake showed a progressively increasing trend, which is potentially attributed to increased metabolic demands resulting from tumor growth and increased invasiveness. For the subtypes, SPH type I potentially exhibited low FDG uptake (52, 53). It may be because of the low invasiveness and/or the volume effect of cystic components, which lead to a reduction in the density of metabolically active cells (20). There was a positive correlation between FDG uptake and the thickness of the cyst wall as well as the presence of wall nodules. The minimum diameter for FDG uptake for a wall nodule was 8 mm (28). Therefore, the diagnostic reliability of PET-CT alone for LCCAs is relatively low, and a 6-month follow-up CT examination could be recommended (54) (Table 3).

**TABLE 3:** Correlation between FDG uptake and LCCA subtypes.

LCCA classification		FDG uptake
SPH	Maki–Mascalchi	
I		Low
II	III	Moderate to marked
III	I/II	Depends on nodule size
IV	IV	Moderate to marked

## Molecular characteristics

### Driver gene mutation

Epidermal growth factor receptor (EGFR) has been identified as the predominant driver mutation in LCCAs, followed by Kirsten rat sarcoma viral oncogene homolog (KRAS), which was consistent with the overall data for lung cancer (40, 41, 44, 55). In a study concerning early-stage lung adenocarcinoma, LCCAs accounted for a higher proportion of tumors with positive anaplastic lymphoma kinase (ALK) or rearranged during transfection (RET) rearrangements (56). However, Toyokawa et al. revealed an association between wild-type EGFR status and lung adenocarcinoma adjacent to emphysematous bullae (34). Discrepancies may stem from differences in patient selection because Toyokawa only included patients with emphysematous lungs. No significant association was found between the types of LCCAs and the status of driver gene mutations (41).

### PD-L1 expression

A higher prevalence of programmed cell death ligand 1 (PD-L1) expression was identified in LCCAs than in non-LCCAs (34, 57). This could be attributed to the inclusion of patients with emphysema, wherein PD-L1 expression was triggered by IFN-γ due to inflammation induced by COPD or smoking (58–61). However, it was unclear whether increased PD-L1 expression was also present in patients with other LCCAs without emphysema. The increase in PD-L1 expression implied that LCCAs could potentially be more amenable to immunotherapy (62, 63). For patients with LCCAs exhibiting increased PD-L1 expression, immunotherapy may be considered as a potential treatment option in the future.

## Disease progression

The progression of LCCAs remains a topic of extensive discussion. Currently, various patterns of LCCA formation have been identified and are broadly categorized into the progression of the cyst wall and the cystic airspace.

### Evolution of the cyst wall

This includes (i) thickening of the pre-existing thin-walled cystic airspace [SPH type I (thin-walled type) that progressed to type II (thick-walled type)] (3, 24, 30, 41), (ii) emergence of new nodules [SPH types I or II that progressed to type III (CWN type, cyst with an associated nodule)], and (iii) enlargement or solidification of existing nodules within the cyst wall (13, 22, 64).

### Evolution of the cystic airspace

This includes (i) enlargement, (ii) shrinkage, (iii) the emergence of ground glass nodules, and (iv) the formation of solid nodules within the cystic airspace (39, 41, 45).

**FIGURE 4:**
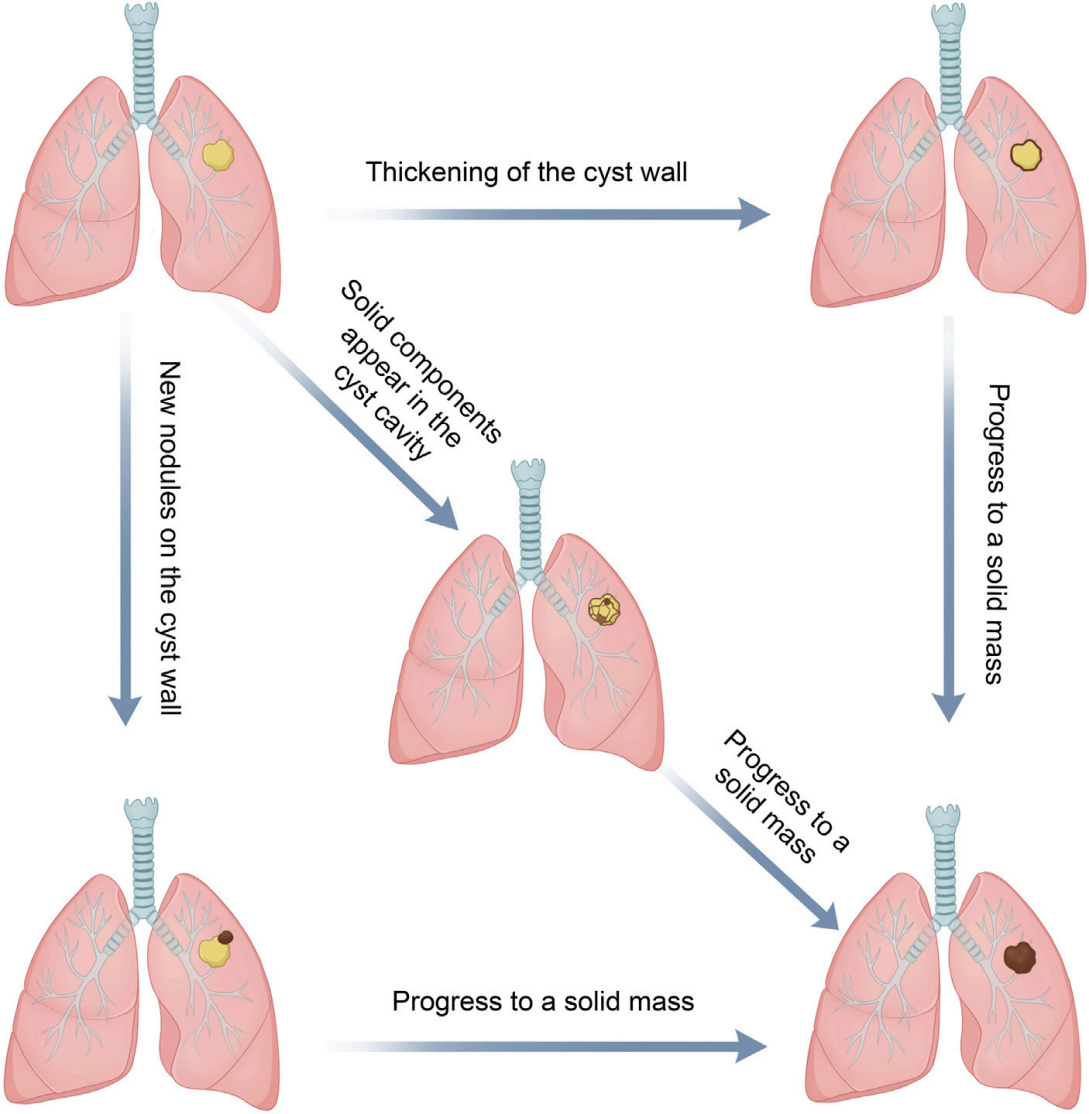
Progression of LCCAs: changes in both the cyst wall and cavity may develop and ultimately culminate in a solid mass.

These transitions might signify tumor progression, whereas changes in the cyst wall and airspace could develop together and ultimately progress to a solid mass (30, 39, 45) (Figure 4). Furthermore, there were some reports of the appearance of new cystic cavities on the basis of solid nodules (53).

The most common evolution of LCCAs was nodule enlargement, followed by the thickening of the cyst wall (20, 40). Changes in the cystic airspaces were more commonly observed as an increase in size, followed by a decrease in size (20). Jung et al. found a commonly observed progression pattern in LCCAs, which was characterized by the gradual enlargement of cysts followed by the emergence of solid nodules. Subsequently, there was a gradual reduction in cyst size accompanied by an increase in the solid component (33). Such progression, particularly the changes of cyst wall and cystic airspaces and nodule development, warranted shorter follow-up intervals or even prompt surgical intervention (21).

The dynamic imaging changes of LCCAs may indicate different follow-up intervals or even shifts in the management strategy. Lung-RADS introduced specific recommendations regarding atypical pulmonary cyst in 2022, with classifications that largely correspond to the SPH classification. According to Lung-RADS v2022 (v 2.0), thin-walled cysts (wall< 2 mm) are recognized as benign; however, thick-walled cysts (2 mm or larger wall thickness), multilocular cysts (thick- or thin-walled cysts with internal septations), and thin- or thick-walled cysts that became multilocular are categorized as 4A, with the recommendation of a 3-month LDCT or PET/CT. In contrast, progressive features such as growing wall thickness or nodularity of the thick-walled cyst, increasing loculation of the multilocular cyst, or new/increasing opacity within or adjacent to the multilocular cyst are upgraded to 4B, for which appropriate diagnostic evaluation is advised. For cysts with mural nodules (either endophytic or exophytic), the higher Lung-RADS category should be assigned, based on the more concerning feature between the cyst wall and the solid component. Although its applicability requires further validation in this specific subgroup, Lung-RADS v2022 provides a framework for managing atypical cystic lesions and is potentially applicable to LCCAs (65).

## Treatment response

Nonoperative management of non-LCCAs generally relies on routine CT follow-up, with the area/volume outperforming the diameter despite practical constraints (66). In contrast, the management of LCCAs requires paying attention to changes in cyst walls, cystic airspaces, and mural nodules for response assessment and follow-up strategy.

### Chemotherapy

The response of LCCAs to chemotherapy was characterized by changes in cystic wall, airspaces, and nodules, which was different from that of non-LCCAs. After neoadjuvant chemotherapy, the solid components of LCCAs exhibited a reduction in size, whereas the cysts either increased or remained unchanged. These changes in the solid constituents might provide a more accurate depiction of treatment response. The enlargement of airspaces may not indicate tumor progression but could reflect treatment-related remodeling, such as the enlargement of check valves secondary to the reduction of solid components. Additionally, alterations in area and volume were more pronounced than changes in diameter, as small linear changes were amplified in two- and three-dimensional measurements (67). Although these parameters may provide a more sensitive reflection of tumor burden, their clinical application remains limited by technical complexity.

Post-treatment CT images clearly demonstrated potential modifications in imaging classification of LCCAs among patients undergoing nonsurgical interventions. However, the impact of post-treatment classification on prognosis remains uncertain, and prospective studies are urgently needed to validate the imaging markers of treatment response in LCCAs, which could ultimately guide personalized management strategies.

### Immunotherapy

Immune checkpoint inhibitors (ICIs) have reshaped the treatment of NSCLC in recent years (68). However, current evidence regarding ICIs in LCCAs is limited to isolated case reports. In a case report by Parisi et al., two patients showed a reduction in the solid component and a thinning of the cyst wall after receiving immune checkpoint inhibitors treatment, which was in line with the tumor response. Another patient whose tumor presented with thickening of the cyst wall and a nodule filling the cyst died 2 months after ICI treatment although the solid target lesion was in a stable state (69). As the current evidence was mainly derived from individual case reports, further prospective studies with systematic in LCCAs are needed to characterize the radiological changes or other imaging markers and their association with the patterns of response of ICIs.

## Survival prognosis

Despite the inherently more aggressive nature, there was no statistically significant difference in disease-free survival (DFS) and overall survival (OS) between LCCAs and non-cystic lung cancer (DFS: type I–IV LCCAs: 100%, 84%, 83%, and 83%, respectively, vs. non-LCCAs: 77%; OS: type I–IV LCCAs: 100%, 84%, 83%, and 83%, respectively, vs. non-LCCAs: 80%) (34, 70). The possible explanation is that early studies excluded some thick-walled-type LCCAs in order to eliminate the bias from cancerous cavities (71).

Micro-papillary components, tumor spread through airspaces, visceral pleura invasion, and lymphovascular invasion were significant predictors of progression-free survival (PFS) in LCCA patients (32, 44). The study by Watanabe et al. revealed that cyst wall thickness served as an independent prognostic factor, with an optimal cutoff point of 4 mm for predicting DFS and OS. In patients of stage I LCCAs, the 5-year OS rate in the thick-walled group was 70.1%, whereas it was 91.5% in the thin-walled group (36). However, these results did not specify whether the measurement of wall thickness encompassed only the solid component or incorporated nonsolid components. Whereas the thick-walled type was associated with a worse prognosis, Ma et al. found that cyst wall thickness and cystic cavity type were independent prognostic factors for LCCA adenocarcinoma subtype but not for squamous cell carcinoma (31). Furthermore, in transcriptomic studies, gene expressions of KCNK3, NRN1, PARVB, and TRHDE-AS1 were associated with the prognosis in adenocarcinomas of LCCAs (44).

Regarding LCCA classification, Shen et al. indicated that there was no statistically significant difference in recurrence-free survival (RFS) between the LCCA and non-LCCA groups. However, SPH type III exhibited a poorer 3-year RFS rate than SPH type I (type I: 100% vs. type III: 77%). In the subgroup analysis, a significant correlation was observed between the volume and diameter of lesions with RFS in SPH type II and III LCCAs (32). The above results were in line with the progression mode of LCCAs.

## Mechanism hypothesis

Currently, there are multiple hypotheses regarding the formation of LCCAs, with the most widely accepted being the check-valve mechanism, in which tumor cells resulted in scar-induced narrowing in small airways, leading to the formation of a valve that causes gas retention and cyst formation (9, 45, 72, 73). This hypothesis was supported by Jung et al., as nonsolid nodules were indicative of cancer cells lining the alveolar wall, potentially leading to the formation of a check-valve (33). Subsequently, the check-valve led to the enlargement of the cyst, which manifested as the presence of cystic components in nonsolid nodules on CT, followed by the emergence of solid nodules at a specific stage. At this point, there was a gradual increase in the solid component that encircled the cyst. Eventually, the cyst shrank (3, 28, 33, 39). This hypothesis could partly explain LCCAs that originated from GGOs.

Another potential explanation was that tumors proliferated along pre-existing lung bullae (14, 28, 45). The presence of emphysematous bullae was a significant risk factor for the development of lung cancer (74–78). The majority of tumors in patients with emphysema were located in close proximity to the emphysematous bullae, and these patients may exhibit increased susceptibility to DNA damage induced by smoking (35). Moreover, chronic inflammation around the emphysematous bullae promoted carcinogenesis and hindered DNA repair processes (70, 78). Pathologically, the cumulative impact of these factors potentially contributed to the increased vulnerability toward carcinogenesis, resulting in the development of lung cancer along the damaged alveolar walls (78).

Regressive changes with absorption in a solid tumor could result from non-necrotic cystic transformation in the solid tumor, thus leading to the formation of cystic airspaces in lung cancer (28, 79). This distinctive feature sets it apart from necrotic cysts exhibiting irregular inner walls.

The pathological findings also provided insights into the etiology of LCCAs. During the progression process, as the cysts enlarged on CT scans, fibrosis developed a thin lining along the boundary of the cystic airspace. With the fibrotic wall becoming progressively thicker and lined with adenocarcinoma cells, the cyst wall gradually appeared thickened. Eventually, infiltrating adenocarcinoma cells gradually invaded the cyst wall while inducing stromal reaction-associated fibrosis. The histopathologic pattern of the tumor could also transform from lepidic and acinar subtypes to solid and micropapillary types, indicating more invasive behavior and poorer prognosis (33).

At the molecular level, transcriptome sequencing revealed upregulation of pathways related to epithelial–mesenchymal transition, angiogenesis, and cell migration in LCCAs compared to non-LCCAs. Additionally, increased immune-suppressive cell infiltration, less B-cell receptor richness, clonality, and high-abundance shared clonotypes were found in LCCAs, which may further deteriorate the antitumor immunity (44). These findings suggest that LCCAs may show distinct mechanisms of development and differences in the immune landscape compared to other types of lung cancer.

## Conclusion

With the widespread adoption of lung cancer screening, the incidence of LCCAs is expected to increase. The radiological classification linked the imaging manifestations of LCCAs with pathological features, and patients with SPH types II–IV required closer surveillance and even needed surgical treatment. Changes in thickened cystic walls and wall nodules, which are indicative of tumor progression, warranted careful consideration. Despite presenting with more invasive features, the overall prognosis of LCCAs did not significantly differ from that of non-LCCAs (32). A diversity of mechanism hypotheses such as check-valve mechanism or progression from pre-existing lung bullae implied the complexity of the etiology of LCCAs. Current diagnostic and therapeutic approaches for LCCAs are limited by the difficulty in distinguishing them from benign lesions during imaging, as well as the uncertainty in grading, staging, and assessing treatment response. Therefore, prospective multicenter studies with long-term follow-up are imperative to validate existing knowledge and standardize the diagnostic and therapeutic strategies for LCCAs.

## Abbreviations

LCCAs, lung cancer associated with cystic airspaces; GGO, ground-glass opacities; SSN, subsolid nodules; EGFR, epidermal growth factor receptor; KRAS, Kirsten rat sarcoma viral oncogene homolog; ALK, anaplastic lymphoma kinase; RET, rearranged during transfection; PD-L1, programmed cell death ligand 1; ICIs, immune checkpoint inhibitors; DFS, disease-free survival; OS, overall survival; PFS, progression-free survival; RFS, recurrence-free survival.
